# A Curated Database of miRNA Mediated Feed-Forward Loops Involving MYC as Master Regulator

**DOI:** 10.1371/journal.pone.0014742

**Published:** 2011-03-03

**Authors:** Mariama El Baroudi, Davide Corà, Carla Bosia, Matteo Osella, Michele Caselle

**Affiliations:** 1 Department of Theoretical Physics, University of Turin and National Institute for Nuclear Physics, Turin, Italy; 2 Center for Complex Systems in Molecular Biology and Medicine, University of Turin, Turin, Italy; 3 Systems Biology Lab, Institute for Cancer Research and Treatment (IRCC), School of Medicine, University of Turin, Turin, Italy; Fondazione Telethon, Italy

## Abstract

**Background:**

The MYC transcription factors are known to be involved in the biology of many human cancer types. But little is known about the Myc/microRNAs cooperation in the regulation of genes at the transcriptional and post-transcriptional level.

**Methodology/Principal Findings:**

Employing independent databases with experimentally validated data, we identified several mixed microRNA/Transcription Factor Feed-Forward Loops regulated by Myc and characterized completely by experimentally supported regulatory interactions, in human. We then studied the statistical and functional properties of these circuits and discussed in more detail a few interesting examples involving E2F1, PTEN, RB1 and VEGF.

**Conclusions/Significance:**

We have assembled and characterized a catalogue of human mixed Transcription Factor/microRNA Feed-Forward Loops, having Myc as master regulator and completely defined by experimentally verified regulatory interactions.

## Introduction

MYC is one of the most intriguing oncogenes and presents the challenging question of how a single gene can manifest so many different effects. There are three Myc gene family members (c-Myc, N-Myc and L-Myc), each with documented oncogenic potential and similar DNA binding properties [1]. They encode basic helix-loop-helix leucine zipper (bHLHZ) transcription factors that are usually found as heterodimers with their obligate partner, the small bHLHZ protein, Max [2]. For simplicity, we will use in the following the term Myc to refer to all the three proteins. Myc Transcription Factors (TFs) may act both as activators or repressors of their Target genes and are involved in a lot of key biological processes, ranging from cell cycle progression to apoptosis and cellular transformation. Our knowledge of Myc transcriptional targets has been greatly enhanced in these last years by a set of high-throughput screenings, which significantly expanded the list of genes that are up or down regulated by Myc [3]–[6]. At the same time it has also been realized that Myc TFs are involved in the regulation of a broad range of microRNAs (miRNAs), many of which have key roles in cell proliferation and oncogenic transformation [7]–[8]. In this respect the Myc family is one of the most interesting examples of TFs exerting at the same time a transcriptional and a (miRNA mediated) post-transcriptional regulation on its targets. It is natural to ask how these two levels of regulation are coordinated and inter-related.

This question is part of a more general effort to understand the interplay between transcriptional and post-transcriptional (miRNA mediated) regulatory interactions which attracted much interest in these last few years [9]–[16].

**Figure 1:**
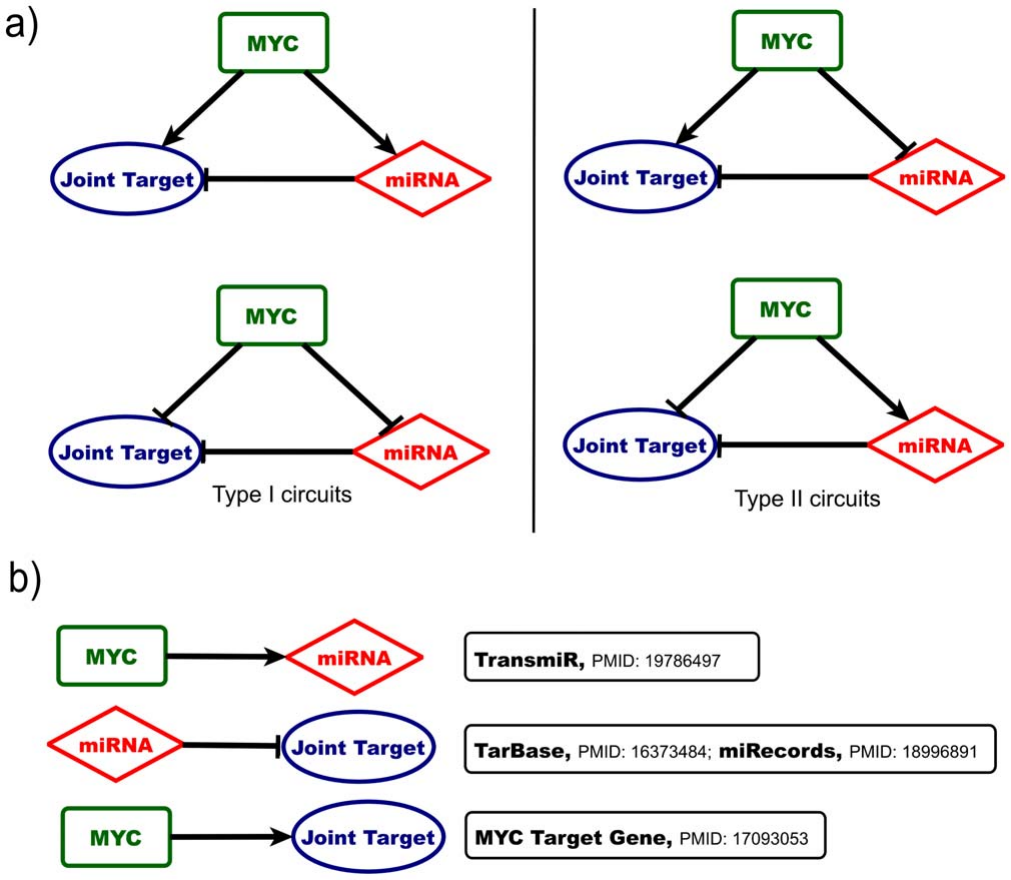
Construction of a catalogue of mixed Feed-Forward regulatory Loops having MYC as master regulator, in which all the regulatory interactions are experimentally validated. a) Representation of a typical mixed single miRNA/Transcription Factor FFL having MYC as master regulator: MYC regulates a miRNA and together with it, a Joint Target protein-coding gene. According to the arrows direction, the mixed FFLs can be classified as incoherent (type I) or coherent (type II) circuits. b) Dataset used for the construction of the mixed FFLs catalogue.

Among the various possible ways to integrate together TF-mediated and miRNA-mediated interactions, a prominent role is played by mixed Feed-Forward Loops (FFLs), in which a master TF regulates a miRNA and together with it a set of protein coding genes that are targeted by the same miRNA. These FFLs, depending on the sign of the regulatory interactions, can be divided in so called incoherent (type_I) or coherent (type_II) circuits [10], [12], [15] (Figure 1). The two types of circuits may lead to very different behaviours. In a typical coherent FFL the miRNA expression is induced by an upstream TF that at the same time represses the transcription of the Joint Target. In this case the miRNA can help the transcriptional repression of a target protein that should not be expressed in a particular cell type, acting as a post-transcriptional failsafe control. Instead, an incoherent FFL can promote high target expression in miRNA-expressing cells, suggesting that miRNAs may have in this case a fine-tuning function, keeping the protein level in the correct functional range and, at the same time controlling the amount of cell to cell fluctuations of the target proteins [10], [17]–[18].

In a recent paper [12] we proposed a bioinformatic pipeline, mainly based on an ab-initio sequence analysis for a genome wide study of this class of mixed FFLs. We could obtain in this way a total of 5030 different single target circuits (corresponding to 638 merged FFLs) in human. Using these data and various randomization techniques we could show that these mixed FFLs are remarkably over-represented in the regulatory network. The major drawback of that analysis was that it was based only on bioinformatic evidences and thus was in principle biased by our assumptions on TF and miRNA binding sequences. The main goal of this paper is to overcome this limitation by constructing a set of mixed FFL in which all the regulatory links were supported by experimental evidences and then use this set to perform unbiased statistical and functional enrichment analysis.

To this end we concentrated our attention on the Myc TF and built a curated database of human mixed miRNA/TF FFLs having Myc as master regulator (Figure 1a) and characterized only by experimental supported regulatory connections (Figure 1b). This choice had two main motivations. The first is that, as we discussed above, Myc is a TF of crucial importance in several biological processes. The second is that for this particular TF a lot of experimental data exist thus allowing us to construct a database large enough to perform reliable statistical and functional analysis.

## Results and Discussion

In this section we report a list of mixed Myc/miRNA FFLs in which all the regulatory interactions are experimentally validated, and a set of statistical and functional tests to show the over-representation of the FFLs in the globally regulatory network. Then, as an example of use of our database, we shall discuss a few FFLs which we found of particular biological relevance and in which the interplay between transcriptional and post-transcriptional regulation seems to play a special role.

### Construction of a list of mixed MYC/miRNA Feed-Forward Loops in which all the regulatory interactions are experimentally validated

Merging together three datasets of experimentally verified Myc-driven interactions and miRNA/target relationships (see Figure 1b and Materials and Methods for details) we were able to identify 110 independent mixed FFL, involving 23 miRNAs regulated by Myc and a total of 71 Joint Target genes (the number of FFLs being larger because a few genes are targeted by more than one miRNA) and among them there were 31 Joint Target genes validated by low throughput experiments.

In addition to that, a manual survey of the published literature allowed us to recognize 33 additional FFLs summing up to a total of 143 FFLs involving 29 Myc regulated miRNAs and 87 Joint Target genes. Out of these 143 FFLs, 26 could be classified as incoherent (type_I), 46 as coherent (type_II) and the remaining 71 could not be associated to any of the two types.

Our results are collected in Supplementary Table S1, where we listed all the FFLs and reported for each of them the corresponding characterization and the PUBMED link for each regulatory interaction. In addition to that, in Supplementary Table S2 we listed the complete dataset of open and closed circuits obtained with our analysis. For completeness, we also included in Supplementary File S1 the list of miRNA/target gene interactions from TarBase and miRecords associated to the miRNAs not targeted by Myc.

**Figure 2:**
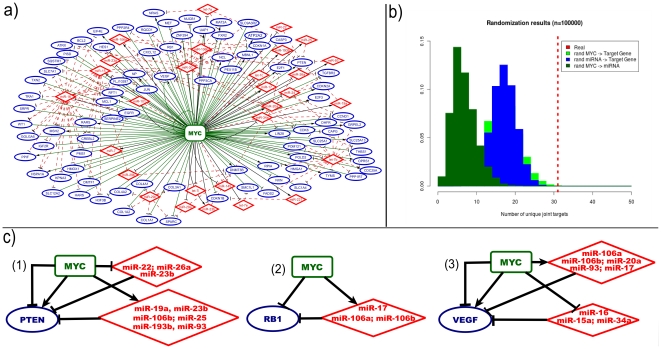
Properties of the mixed FFLs. a) Graphical representation of the network obtained combining together all the MYC-centred mixed FFLs. MYC is depicted in green, nodes in red (diamonds) correspond to miRNAs, whereas the blue ones (ellipses) correspond to Joint Targets. Biological relationship between two nodes is represented as an edge (edges in green identify targets regulation by Myc, black edges evidence the miRNAs regulation by Myc and in red the targets regulated by miRNAs). b) Randomization results for the over-representation analysis of Myc induced mixed FFLs validated with low throughput experiments. We plotted the number of Joint Target genes obtained in the real Myc network, alongside the distributions (normalized histograms) of the number of Joint Target genes detected in the three randomization strategies adopted c) A few interesting examples of FFLs, having as Joint Target: (1)PTEN, (2)RB1 and (3)VEGF.

In Figure 2a we reported a graphical representation of the resulting network of Myc-centred mixed FFLs database. In addition, we have recently released an user friendly web server, CircuitsDB - as interface to the catalogues of computational predicted mixed FFLs, in human and mouse that we previously developed [12], [24]. We added a new section to CircuitsDB devoted to the MYC FFL, thus allowing an user to browse on-line our data and to follow the miRNAs and Target gene annotations. CircuitsDB is freely available at http://biocluster.di.unito.it/circuits.

### Statistical and Functional analysis of the MYC/miRNAs Regulatory Network

#### Mixed FFLs over-representation

As discussed in the introduction, one of the aims of our work was to use our dataset to test in an unbiased way the over-representation of mixed FFLs in the Myc/miRNA regulatory network. This is the reason for which in the previous section we separated the manually curated FFLs (the last 33) from those obtained performing an unbiased intersection of Myc/target and miRNA/target (TarBase and miRecords) databases. To test if the FFLs are over-represented, we performed an independent permutation test for each link of the FFL. In order to eliminate a possible experimental bias in this test, we filtered the data from TarBase and miRecords keeping only miRNA/Target gene interactions inferred by low throughput experiments. In all the three tests we found a significant enrichment of our FFLs with respect to the random samples. We report the results of this analysis in Figure 2b. To show the statistical significance we calculated the Z-score (Z) of the number of Joint Target genes in the closed circuits for each randomization (MYC → Target Gene reshuffling: Z = 3.5; miRNA → Target Gene reshuffling: Z = 4.5 and MYC → miRNA reshuffling: Z = 7.7). A detailed description of the tests can be found in the Supplementary Material S1 (section S3). We also performed the same test considering all the miRNA/target gene interactions from miRecords and TarBase. Also for these tests, results and details are reported in Supplementary Material S1 (section S3, Figure S1 and Figure S2).

#### Mixed FFLs redundancy

Looking at Table S1 and Figure 2a it is easy to see that a remarkable feature of the Myc-centred FFL network is that, at least for a few FFLs, it is strongly redundant on the miRNA side. Moreover this redundancy seems to be associated to targets, like PTEN, VEGF, E2F1 or Myc itself, which are all master regulators playing a major role in several biological processes. It is important to notice that this redundancy is highly non trivial since it is not simply due to miRNAs belonging to the same cluster but instead involves miRNAs belonging to different Transcriptional Units [25] and characterized by different “seeds”.

It is difficult to assign a statistical significance to this redundancy since it is obviously biased by the relevance of the targets, but we guess that it is not random and was selected by evolution to enhance the robustness and flexibility of these circuits.

#### Functional annotations

The targets of the mixed Myc-centred FFLs show a remarkable enrichment in functional categories involved in cell cycle regulation and in various types of cancer (see Table 1).

**Table 1:** Functional Annotation Chart, performed using DAVID Bioinformatics tool.

KEGG pathways	Genes	p-Value	Benjamini (corrected p-value)
Pathways in cancer	BCL2, E2F1, E2F3, CASP3, EGFR, VEGF, COL4A1, COL4A2, CD1, CDK6, p21, p27, CDKI2A, JUN, MET, MSH2, RAS, PTEN, RB1, TGFb2, MYC	3.8E-11	1.8E-9
Bladder cancer	E2F1, E2F3, EGFR, CD1, CDKI2A, CDKI1A, RAS, RB1, VEGF, MYC, THBS1	8.7E-12	8.4E-10
Chronic myeloid leukemia	E2F1, E2F3, CD1, CDK6, CDKI1A, CDKI1B, CDKI2A, RAS, RB1, TGFb2, MYC	3.7E-9	9.1E-8
Small cell lung cancer	BCL2, E2F1, E2F3, COL4A1, COL4A2, CD1, CDKI1B, CDK6, PTEN, RB1, MYC	1.2E-8	2.3E-7
Melanoma	E2F1, E2F3, CD1, CDKI2A, CDKI1A, CDK6, EGFR, RAS, RB1, PTEN, MET	2.2E-9	7.0E-8
Glioma	E2F1, E2F3, CD1,CDKI2A, CDKI1A, CDK6, EGFR, RAS, RB1, PTEN	1.3E-8	2.1E-7

GO Terms	Genes	p-Value	Benjamini (corrected p-value)
Response to abiotic stimulus	BCL2, CASP3, CD1, CDKI1A, COL1A1, COL3A1, DNMT3B, EGFR, MAT2A, JUN, MSH2, SLC12A2, RAS, SMC1L1, THBS1, TGFb2, MYC	5.3E-10	8.6E-7
Regulation of cell cycle	BCL2, E2F1, EGFR, CASP3, CD1, CDK6, CDKI1A, CDKI1B, CDKI2A, CDC25A, JUN, MSH2, PTEN, SMC1L1, RB1, MYC	1.1E-9	4.5E-7

We report the Gene Ontology (GO) Terms and KEGG pathways Over-represented among Joint Target Genes, performed using DAVID Bioinformatics tool. For each row the corresponding Benjamini (corrected for multiple testing) as well as the raw hypergeometric p-values are indicated.

It is important to notice that this enrichment is indeed a general property of the genes targeted by the miRNAs under the control of Myc (see Table S3 and the discussion in the supplementary section S4). What is remarkable is the fact that this enrichment is somehow concentrated in the set of FFLs targets and seems to disappear if the same analysis is performed on the targets of the open circuits (see Table S3).

#### miRNA/Target co-localization and co-regulation

A mandatory condition for FFLs to play a functional role in the cell is the simultaneous presence in the same tissue of the genes and miRNAs involved in the FFL. Even if we concentrated in our work only on experimentally validated interactions, this does not automatically guarantee that the FFL is actually realized in a given tissue. In order to address this issue, we included in our on-line database a link to a set of tissue specific microarray results which can be visualized as a heat map. This tool allows a qualitative inspection of co-regulation and of a possible involvement of the various players of the FFL in the same biological context.

### Discussion of a few interesting circuits

The best known example of mixed FFL is the c-Myc/E2F1/miR-17∼20a circuit which was discussed for the first time in [26] and since then has been the subject of several works. Looking at our database we are able to show that this is indeed a redundant FFL and that E2F1 is also regulated by miR-106b which acts coherently with miR-17∼20a (even if it belongs to another Transcriptional Unit). This shows that the complex regulatory network joining c-Myc and E2F1 [27]-[28] is even more intricate than expected.

As further examples of the use of our database and of the type of biological insight that one can reach combining transcriptional and post-transcriptional regulation, we shall discuss in this section three other FFLs in which the single regulatory interactions where all known in the literature but their common involvement in a closed FFL was not realized before.

### 1) The MYC/PTEN/miR-106b, miR-93, miR-25, miR-19a, miR-22, miR-26a, miR-193b, miR-23b circuit

Pten (Platelet-derived endothelial cell growth factor) is a tumour suppressor gene which plays an important role in various cancer related pathways. PTEN is known to be the target of several miRNAs [29]-[31] and, remarkably enough, most of these miRNAs are under the control of Myc thus closing a set of parallel FFLs (Figure 2c (1)).

Of particular interest is the incoherent branch of the circuit, which is mediated by miR-19a, and by the miR-106b-25 cluster (via the activation of the hosting gene MCM7).

Following our recent analysis [17], we suggest that this particular type of FFL should act as a noise buffering circuit and should guarantee a steady level of the PTEN protein. Indeed it was recently shown [32] that even subtle variations in the expression of PTEN are sufficient to promote cancer susceptibility. This supports the idea that this circuit could have been selected by evolution to ensure stability of PTEN levels against fluctuations of upstream regulators.

### 2) The MYC/RB1/miR-106a, miR-106b and miR-17 circuit

The circuit in Figure 2c (2) plays a key role in cancer pathogenesis of solid cancer, by controlling the expression of the retinoblastoma protein (*RB1*) which is a tumour suppressor and was shown to be dysfunctional in many types of cancer.

It is a coherent FFL since Myc represses the transcription of RB1 and at the same activates a set of miRNAs which in turn inhibit the translation of RB1 [31]. As mentioned in the introduction it is likely that coherent FFLs like this one should play the role of post-transcriptional failsafe controls on their targets. This prediction seems to be in good agreement with the results of [33] where a detailed study of the miR-106a/RB1 regulatory interaction in colon cancer cells is reported and a failsafe control role of this miRNA on the level of RB1 is suggested.

### 3) The MYC/VEGF/miR-106b, miR-106a, miR-93, miR-34a, miR-20a, miR-17, miR-16, miR-15a circuit

The Vascular endothelial growth factor (VEGF), is one of the most important angiogenic growth factors, and is involved in a host of biological processes ranging from cell migration to apoptosis. VEGF translation is strictly controlled by at least eight miRNAs and, as for PTEN, all of these miRNAs are under the control of Myc, thus closing a highly redundant FFL. The circuit in Figure 2c (3) can be classified as coherent or incoherent loop, depending on the action of Myc on its targets.

It would be very interesting to understand if the different topologies which this FFL may assume, depending on the type of regulation exerted by Myc, may be associated to the different functional roles of VEGF.

### Conclusion

It has become by now clear that the interplay between transcriptional and post-transcriptional (miRNA mediated) regulation plays a crucial role in the modulation of gene expression. Up to a few years ago this issue could be addressed only with bioinformatic tools. Remarkable results were obtained in this way but they were unavoidably affected by large numbers of false positives thus making impossible to assess their validity with reliable statistical tests and yielding at the same time very challenging the experimental validation of the predicted interactions. In these last few years thanks to the impressive improvement of high-throughput technologies, larger and larger databases of validated interactions appeared, thus making it possible to construct networks of validated interactions of increasing complexity. In this paper we in particular concentrated on the mixed network centred on the Myc Transcription Factor.

While it is likely that in the near future further links will be added to this network due to new experiments, those which are already known in the literature allowed us to draw a rich and intricate picture of the Myc-centred mixed regulatory network, which turns out to be strongly enriched in mixed FFLs.

As mentioned above, in several cases we could also fix the relative sign of the various regulatory interactions in the FFLs so that we could distinguish between coherent and incoherent FFLs.

It is important to stress that these two classes of FFLs may lead to very different behaviours [10], [15]. The coherent circuits lead to a reinforcement of the transcriptional regulation at the post-transcriptional level and might be important to eliminate the already transcribed mRNAs when the transcription of a Target gene is switched off. The incoherent circuits can be used to stabilize the steady state production of a protein by dumping transcriptional fluctuations. In a simple TF/Target interaction, any fluctuation of a master TF could induce a non-linear increase in the amount of its target products. The presence, among the targets, of a miRNA that down-regulates the other targets might represent a simple and effective way to control these fluctuations.

It is interesting to notice that from our data these two classes of FFLs seem to be enriched in a substantially equivalent way, thus suggesting that both functions, post-transcriptional failsafe control and noise reduction are of crucial importance for higher eukaryotes and are effectively implemented by miRNA mediated regulation.

At the same time these results allowed us to improve our understanding of a few non-trivial issues like the remarkable stability of PTEN levels in normal cells, which could be a consequence of the incoherent FFL targeting this gene or the high levels of redundancy of FFLs targeting master regulators, which could be a way to ensure tight but at the same time flexible regulation of these master genes.

A future challenge will be to identify systematically new mixed FFL involving others important Transcription Factors and to understand their biological function, integrating together the wealth of experimental data that will be available in the next years. Under the hypothesis that FFLs are over-represented in the global Myc/miRNAs network, they could have an important role in stabilizing gene expression levels against external noise sources [10], [15], [17]. It could be of interest to study the noise properties of the different FFLs and their functional importance.

## Materials and Methods

The construction of the curated database of mixed FFLs, having Myc as master regulator was mainly based on the following independent resources (Figure 1b):

(Details on all these steps of the analysis can be found in sections S1-S4 of Supplementary Material S1)

1. MYC → miRNA link: 26 interactions taken from the *TransmiR* database [19] plus 20 additional miRNA induced by Myc from manual literature search.
2. miRNA → Target Gene link: data taken from the *TarBase* database, v. 5.0 [20] and from the *miRecords* database, v. 1.0 [21] for a total of 1276 non redundant interactions.
3. MYC → Target Gene link: data taken from the *Myc Target Gene* database [22] comprising a total of 1733 interactions.

The list of Myc-centered mixed FFLs, was then obtained by merging together the above databases. Random reshufflings of miRNAs and gene names in the original transcriptional (MYC → Target Gene and MYC → miRNA) and post-transcriptional (miRNA → Target Gene) regulatory networks were performed to assess the statistical significance of the over-representation of the mixed Myc/miRNA FFLs in the global Myc/miRNA regulatory network.

To investigate whether the Joint Target genes belonging to our catalogue of mixed FFLs could be associated to specific biological function, we looked for GO terms and KEGG pathways enrichment analysis among them, using the DAVID Bioinformatics tools [23].

## Supporting Information

Supplementary Material S1 Supplementary Material Sections and References: S1) Description of the databases used for the construction of the mixed FFLs; S2) Identification of additional mixed FFLs; S3) Additional information on the over-representation test; S4) Additional information on the functional enrichment test; References.
(0.05 MB DOC)

Table S1 List of the MYC centred mixed FFLs having the single links experimentally validated with their corresponding PMID references. The table lists the 143 mixed FFLs, having Myc as master regulator and characterized only by experimentally verified interactions. Each line corresponds to a single closed FFL. For the Joint Target genes, we used the standard HGNC identifiers and the Ensembl gene id. For the miRNA, stable ids from the miRBase database. The type of regulation exerted by Myc on miRNA and Joint Target is indicated, respectively, with Myc_Action 1 and Myc_Action 2. There are PMID references for each link: Reference 1: refers to Myc/miRNA interaction, Reference 2: refers to miRNA/Joint Target interaction and Reference 3: refers to Myc/Joint Target interaction. We highlighted with (§) symbol additional FFLs from manual literature search and with (*) symbol low throughput validation of miRNA/target genes interactions reported in TarBase or miRecords databases.
(0.04 MB XLS)

Table S2 List of all open and closed circuits. The table lists the 565 open and closed circuits having Myc as master regulator and characterized only by experimentally verified interactions. Each line corresponds to a single circuit. For the Joint Target genes, we used the standard HGNC identifiers and the Ensembl gene id. For the miRNA, stable ids from the miRBase database. The following symbols indicate: (*) low throughput validation of miRNA/target genes interactions reported in TarBase and miRecords databases; if miRNA/target genes interactions were reported in Tarbase or miRecords (+) and if these interactions were found in literature (−).
(0.07 MB XLS)

Table S3 Functional Annotation Chart, performed using DAVID Bioinformatics tool. We report only the KEGG pathways discussed in the main text (see Table 1). We compare the enrichment levels of the FFL targets, the 316 genes targeted by the miRNAs which are under the control of Myc and the 662 genes targeted by miRNAs which are not under the control of Myc.
(0.01 MB XLS)

File S1 The list of 662 targets associated to the miRNAs not targeted by Myc. For the target genes, we used the standard HGNC identifiers and we highlighted with (*) the miRNA/target genes interactions experimentally directly validated. For the miRNA, stable ids from the miRBase database.
(0.01 MB TXT)

Figure S1 Distribution of miRNA target genes. We organized miRNAs in four classes, based on the number of miRNA target experimentally validated genes interactions.
(0.02 MB PNG)

Figure S2 Randomization results for the over-representation analysis of Myc induced mixed FFLs experimentally validated by low and high throughput experiments. We plotted the number of Joint Target genes obtained in the real Myc network, alongside the distributions (normalized histograms) of the number of Joint Target genes detected in the three randomization strategies adopted.
(0.02 MB PNG)
