# Supplementary material for: A Curated Database of miRNA Mediated Feed-Forward Loops Involving MYC as Master Regulator
Source: PLoS One. 2011 Mar 3;6(3):e14742. doi: 10.1371/journal.pone.0014742 (PMC3048388; doi:10.1371/journal.pone.0014742)
Supplement: Supplementary Material S1 — Supplementary Material Sections and References: S1) Description of the databases used for the construction of the mixed FFLs; S2) Identification of additional mixed FFLs; S3) Additional information on the over-representation test; S4) Additional information on the functional enrichment test; References. (0.05 MB DOC) [file pone.0014742.s001.doc]

**A curated database of miRNA mediated Feed Forward Loops involving MYC as Master Regulator**

**Mariama El Baroudi^1,2,*^, Davide Corà^2,3,*^, Carla Bosia^1,2^, Matteo Osella^1,2^, Michele Caselle^1,2^**

**1** Department of Theoretical Physics, University of Turin and National Institute for Nuclear Physics*,*Turin, Italy, **2** Center for Complex Systems in Molecular Biology and Medicine, University of Turin, Turin, Italy, **3** Systems Biology Lab, Institute for Cancer Research and Treatment (IRCC), School of Medicine, University of Turin, Turin, Italy, ***** These authors contributed equally to this work.

**Supplementary Material**

**S1: *Description of the databases used for the construction of the mixed FFLs***

Here, we describe the different resources used for the construction of our database of Myc-centered mixed Feed-Forward Loops (FFLs) separately for each link:

MYC 🡪 Target Gene

The Myc Target Gene database [1] contains genes responsive to the Myc Transcription Factor (TF), according to experimental evidence reported in literature. It reports a total of 1733 targets. Interestingly, if available, the database also reports the type of regulation exerted by Myc on the target gene (up_regulation or down_regulation).

MYC 🡪 miRNA

The *TransmiR* database is a collection of TF/microRNA (miRNA) regulations, based only on experimental evidences. The authors manually surveyed approximately 5000 reports in literature and identified 243 TF - miRNA regulatory relationships, which were supported experimentally from 86 publications (TransmiR v. 1.0). They then used these data to build a TF/miRNA regulatory database (TransmiR, http://cmbi.bjmu.edu.cn/transmir), which contains, focusing on the human case, a total of 222 regulatory interactions among 72 TFs and 91 target miRNAs. Among these TFs, there is also Myc (quoted as Myc or MycN), which is reported to regulate 26 different miRNAs [2]. If available, the database also contains the type of regulation exerted by Myc on the miRNA. In all the cases, the reported TF/miRNA interactions can be traced back, via the PUBMED id, to the original publication in which the interaction is reported.

We added to these 26 miRNAs targeted by Myc a few others which we found in the literature and were not present in TransmiR. We listed them here together with their Pubmed reference:

- miR-145 [3].
- miR-24 [4].
- miR-106b-25 [5].
- miR-7, miR-30, miR-26b, miR-146a, miR-150, miR-29b, miR-497, miR-99a [6].
- miR-193b, miR-222 and miR-98 [7].
- miR-9 [8].

Finally, we included in our list also the intronic miRNAs clusters mir-218-2, mir-326, mir-101-2, mir-149, mir-25, hosted respectively in the SLIT3, ARRB1, RCL1, GPC1, MCM7 genes which are Myc targets.

We found in this way a total of 46 miRNAs under the control of Myc.

As we shall see in the next section, only for 41 out of these 46 miRNAs it is possible to find in the published literature experimentally validated targets (Table S2). Only 29 were found to participate in at least one closed circuit (Table S1).

miRNA 🡪 Target Gene

*TarBase* [9] is a database of experimentally validated miRNA target gene interactions. It contains 108 human miRNAs, regulating 858 protein-coding genes, with 1025 miRNA/target interactions (TarBase v.5.0), which can be grouped according to the type of the experimental evidence supporting the miRNA/target interaction. In all the cases, the interactions can be traced back, via the PUBMED id, to the original publication in which they were reported. In parallel to that, in a recent work, the authors of *miRecords* [10] tried to establish a centralized resource aimed at the unification of the predictions of several different miRNA target genes interactions databases. In particular, their work also contains a section devoted to experimentally validated data reported from direct inspection of the literature, resulting in 103 human miRNAs, 597 target genes and 768 miRNA/target interactions (miRecords v. 1.0). Combining the two datasets, we obtained a list of experimentally validated miRNA/target interactions, containing 110 miRNAs, 986 target genes and a total of 1276 independent interactions and 434 of these interactions were validated by low throughput experiments. Among these miRNAs we are in particular interested in the 37 targeted by Myc included in the 41 discussed above (summing also the results of the previous section, for a total of 9 of the miRNAs regulated by Myc we could not find any target in the databases of experimentally validated miRNA/target interactions and they are thus excluded in the following analysis). We recognized that, according to the current versions of TarBase and miRecords, these 37 miRNAs regulate a total of 380 protein coding genes and we found 93 of them validated by low throughput experiments, which were included and highlighted with (*) symbol in Supplementary material Table S2.

**S2: *Identification of additional mixed FFLs***

As mentioned in the main text, combining together the above datasets we were able to find 110 mixed FFLs involving a total of 71 Joint Target genes.

In order to obtain the largest possible collection of Myc centered FFLs we manually tested the 309 miRNA targets (out of the 380 mentioned above) which were not already involved in FFLs, looking for possible evidences of Myc regulation not reported in the Myc Target Gene database (www.myc-cancer-gene.org/). To this end, besides a direct search in the literature, we used the Transfac_professional database [11] and the dataset of Myc-regulated gene targets, resulting from a genome-wide, unbiased characterization in a model of human B cells, using Chromatin Immunoprecipitation coupled with pair-end ditag sequencing analysis (ChIP–PET) [12].

In this way we could find 15 additional Myc-regulated genes: ATP2A2, CAPG, DNMT3B, GRPEL2, LIN28, MBNL1, NP, OPRS1, PISD, RARS, RFT1, SLC25A1, SLC25A13, TXN2 and ZNF294 which we could associate to 32 new FFLs. In addition to that, we further added a FFL involving CASP3 as Joint Target found to be regulated by hsa-let-7a in literature[13], summing up to a total of 143 FFLs involving 29 Myc regulated miRNAs and 87 Joint Target genes (Table S1). For completeness, we also included in Supplementary File S1 the list of miRNA/target gene interactions from TarBase and miRecords associated to the miRNAs not targeted by Myc.

**S3: *Additional information on the over-representation test***

In Figure 2b we reported the results of the randomization test considering only miRNA/target genes interactions validated by low throughput experiments, to test the over-representation of the FFLs in the global Myc/miRNAs regulatory network.

We chose three different types of randomization strategies based on unbaised random reshuffling of miRNA and gene names in our databases. Each randomization test was performed 100000 times and we evaluated their statistical significance by calculating a Z-score (Z) (reported in the main test).

- In the first test (MYC 🡪 Target Gene reshuffling): we generated a total of 1733 putative MYC regulated genes by randomly assigning their target genes from the total amount of Known protein coding genes.
- In the second test (miRNA 🡪 Target Gene reshuffling): we extracted randomly 93 putative miRNA target genes from all the non-redundant union of the TarBase and miRercords databases validated by low throughput experiments.
- In the last test (MYC 🡪 miRNA reshuffling): we picked up randomly 37 miRNAs from the non-redundant union of the TarBase and miRercords and then extracted the corresponding target genes (selecting only those validated by low throughput experiments).

We then used these randomized sets to rebuild our dataset of mixed Myc FFLs and we compared the number of Joint Targets in the random datasets with the number of Joint Target present in the real mixed Myc FFLs (Figure 2b).

Finally, as a further test, we performed the same analysis using the set of all the miRNA/target experimentally validated genes interactions from miRecords and TarBase (i.e. we included also those obtained with high-throughput experiments).

In the case of Myc 🡪 miRNA reshuffling the number of validated targets was large enough to allow us to introduce an additional improvement in this permutation test. We organized the miRNAs (both those which are Myc targets and those which are not Myc targets) in four classes on the basis of the number of their targets and performed the reshuffling only inside each class. In this way we tried to keep into account the fact that not all the miRNAs are on the same ground, but there are relevant differences in their regulatory role inside the cell, and in particular in the number of targets. We selected the four classes looking at the distribution of targets (which is plotted in Figure S1), fitting with an approximate exponentially decreasing law.

The results of our reshuffling test are reported in Figure S2. The corresponding Z scores are (MYC 🡪 Target Gene reshuffling: Z = 8.2); (miRNA 🡪 Target Gene reshuffling: Z = 3.6) and (MYC 🡪 miRNA reshuffling: Z = 2.4).

**S4: *Additional information on the functional enrichment test***

To investigate whether the Joint Target genes belonging to our catalogue of mixed FFLs could be associated to specific biological function, we looked for GO terms and KEGG pathways enrichment analysis among them, using the DAVID Bioinformatics tools [14]. The results are reported in Table 1.

However, as mentioned in the main text, the p-values reported in Table 1 must be taken with some caution. In order to give a more precise meaning to these p-values we decided to analyse with the same protocol also the targets of open and closed circuits, the targets of open circuits and then the targets of miRNAs not regulated by Myc (Table S3). In these two control studies we concentrated only on the cancer related KEGG pathways listed in Table 1. We report the results in Table S3. As it is easy to see the involvement in cancer related pathways seems to be a general feature of the targets of the miRNAs under the control of Myc. It is very interesting to notice the large jump in p-values between these targets and those of the miRNAs not under the control of Myc. Since the comparison between the two samples is, as far as we can see, unbiased (they are more or less of the same size and are extracted from the same database) we consider this results rather robust and reliable.

**References:**

1. Zeller KI, Jegga AG, Bruce J, Aronow BJ, Kathyrn A, et al. (2003) An integrated database of genes responsive to the Myc oncogenic transcription factor: identification of direct genomic targets. *Genome Biology* **4:** R69.

2. Wang J, Lu M, Qiu C, Cui Q (2009) TransmiR: a transcription factor–microRNA regulation database. *Nucleic Acids Res* **1:** 4.

3. Sachdeva M, Zhu S, Wu F, Wu H, Walia V, et al. (2009) p53 represses c-Myc through induction of the tumor suppressor miR-145. *Proc Natl Acad Sci* USA **106:** 3207-3212.

4. Gao P, Tchernyshyov I, Chang TC, Lee YS, Kita K, et al. (2009) c-Myc suppression of miR-23a/b enhances mitochondrial glutaminase expression and glutamine metabolism. *Nature* **458:** 762–765.

5. Petrocca F, Visone R, Onelli MR, Shah MH, Nicoloso MS, et al. (2008) E2F1-regulated microRNAs impair TGFbeta-dependent cell-cycle arrest and apoptosis in gastric cancer. *Cancer Cell* **13:** 272–286.

6. Chang TC, Yu D, Lee Y-S, Wentzel EA, Arking DE, et al. (2008) Widespread microRNA repression by Myc contributes to tumorigenesis. *Nat Genet* **40:** 43–50.

**7.** Kim **JW, Mori S,** Nevins **JR (2010) Myc-induced microRNAs integrate Myc-mediated cell proliferation and cell fate.** Cancer Res **70: 4820-8.**

**8. Khew-Godall Y, Goodall GJ (2010) Myc-modulated miR-9 makes more metastases. *Nat cell Biol* 12: 209-211.**

**9. Papadopoulos GL, Reczko M, Simossis VA, Sethupathy P, Hatzigeorgiou AG (2009) The database of experimentally supported targets: a functional update of TarBase. *Nucleic Acids Res* 37: D155-D158.**

**10. Xiao F, Zuo Z, Cai G, Kang S, Gao X, et al.** (2009) miRecords: an integrated resource for microRNA–target interactions. *Nucleic Acids Res* **37:** D105-D110.

11. Matys V, Kel-Margoulis OV, Fricke E, Liebich I, Land S, et al. (2006) TRANSFAC and its module TRANSCompel: transcriptional gene regulation in eukaryotes. *Nucleic Acids Res* **34:** D108-110.

12. Zeller KI, Zhao X, Lee CW, Chiu KP, Yao F, et al. (2006) Global mapping of c_Myc binding sites and target gene networks in human B cells. *Proc Natl Acad Sci U S A* **103**: 17834-17839.

13. Tsang WP, Kwok TT (2008) Let-7a microRNA suppresses therapeutics-induced cancer cell death by targeting caspase-3. *Apoptosis* **13:** 1215-1222.

14. Huang DW, Sherman BT, Lempicki RA (2009) Systematic and integrative analysis of large gene lists using david bioinformatics resources. *Nat Protoc* **4**: 44–57.
