# Supplementary figures and images for: A Curated Database of miRNA Mediated Feed-Forward Loops Involving MYC as Master Regulator
Source: PLoS One. 2011 Mar 3;6(3):e14742. doi: 10.1371/journal.pone.0014742 (PMC3048388; doi:10.1371/journal.pone.0014742)

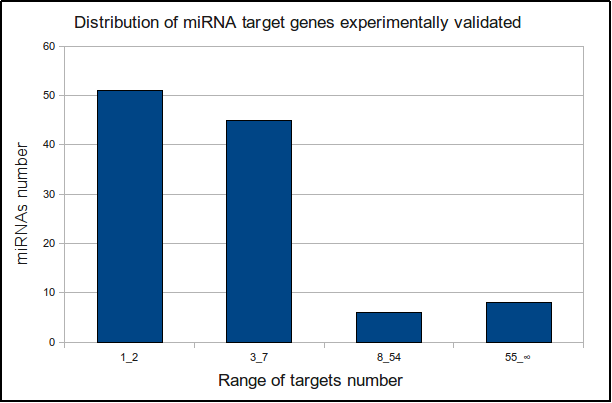

Supplement: Figure S1 — Distribution of miRNA target genes. We organized miRNAs in four classes, based on the number of miRNA target experimentally validated genes interactions. (0.02 MB PNG) [file pone.0014742.s006.png]

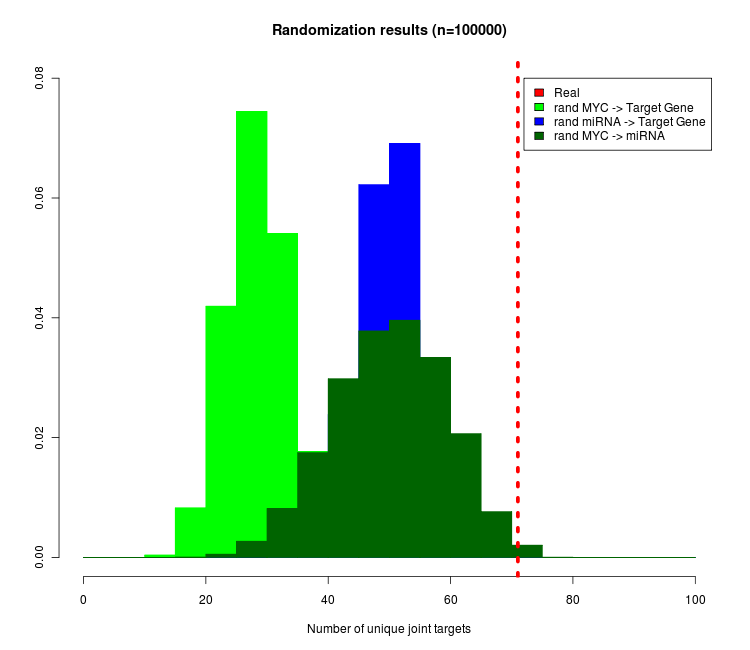

Supplement: Figure S2 — Randomization results for the over-representation analysis of Myc induced mixed FFLs experimentally validated by low and high throughput experiments. We plotted the number of Joint Target genes obtained in the real Myc network, alongside the distributions (normalized histograms) of the number of Joint Target genes detected in the three randomization strategies adopted. (0.02 MB PNG) [file pone.0014742.s007.png]
